# Editorial for “Common data elements for maternal health research: Expert consensus emerging from a modified Delphi study”

**DOI:** 10.1002/pmf2.70126

**Published:** 2025-10-09

**Authors:** Sarah E. White, David M. Stamilio

**Affiliations:** ^1^ Department of Obstetrics and Gynecology Maternal Fetal Medicine Division Wake Forest University School of Medicine Winston Salem North Carolina USA; ^2^ Department of Obstetrics and Gynecology Maternal Fetal Medicine Division Department of Epidemiology and Prevention Wake Forest University School of Medicine Winston Salem North Carolina USA

1

Maternal severe morbidity and maternal mortality rates in the United States are unacceptably high and have been increasing in recent years [1, 2]. Notably, the United States has the highest maternal death rate among high‐income countries; there are 22 maternal deaths per 100,000 live births overall, and among US Black women, the death rate is nearly 50 per 1000,000 live births [3]. This represents a two‐ to sevenfold increase compared to most other developed countries. Alarmingly, maternal mortality review committees determined that 84% of pregnancy‐related deaths in the United States were preventable [4]. Accordingly, patient safety initiatives and research designed to mitigate the high risks of maternal morbidity and mortality have been identified as a critical priority by the NIH and other healthcare organizations. However, advancing research to improve maternal health in the United States will require concerted strategies that go beyond the efforts of individual researchers or separate research teams. To advance maternal health science to enable effective prediction and prevention of maternal morbidity and mortality, the national (and really, international) clinical research community must direct efforts in an organized and collaborative manner, with like‐mindedness in data management and sharing, study design and goals, as well as infrastructure and policies for data sharing. One major step toward collaborative research and realizing improved maternal health outcomes is establishing a universally shared approach for data standardization and harmonization.

In this issue of *Pregnancy*, Stierman and colleagues report a study designed to develop such a framework for data standardization and promoting data sharing. Their study aimed to develop a minimum set of priority common data elements (CDEs) for maternal health research using a modified Delphi technique [5]. To accomplish this goal, the Eunice Kennedy Shriver National Institute of Child Health and Human Development convened a panel of experts, including representatives from research centers, professional associations, and government agencies. Using the anonymous, iterative, web‐based, expert consensus‐building Delphi process, the panel produced recommendations to standardize data collection of a minimum set of priority constructs for maternal health research that will aid in advancing maternal health science. Through the rigorous consensus process, they identified a minimum set of 36 biomedical and 22 psychosocial constructs that are prioritized for research, each of which represents a health condition, medical procedure, or behavior. They then developed consensus for how to measure the constructs (i.e., CDEs), including recommended questions and response options for obtaining the data, data sources, and guidance on preferred or alternative approaches for measuring some CDEs.

Presently, the lack of data standardization hampers both clinical practice and research. From a clinical standpoint, variability across studies in exposure and outcome variable definitions and selection makes it difficult to synthesize the body of maternal health research into a coherent summary to establish best practice guidelines [6]. From a health science standpoint, the lack of data standardization hampers the ability to perform valid systematic reviews with unbiased meta‐analysis and limits predictive performance in studies that employ artificial intelligence or machine learning (ML) methods.

In the case of meta‐analysis, heterogeneity in outcomes assessed, outcome measurement methods, and population characteristics across studies included in a systematic review often precludes the ability to pool data or, at the very least, decreases the certainty around pooled estimates due to increased risk for information bias. Too often, studies with heterogeneous data and variable study method quality are meta‐analyzed without adequately assessing or accounting for the heterogeneity. In addition, investigators can select inappropriate outcomes, resulting in an invalid or misleading meta‐analysis due to reporting bias [7]. Establishing vetted CDEs can help mitigate these potential pitfalls of meta‐analysis.

As ML methods are more widely used for clinical prediction, it has become evident that one major barrier to effective ML‐based prediction is the lack of data standardization and completeness in both electronic health records (EHRs) and established research databases [8]. In addition, without established CDEs, a considerable amount of effort is required for each ML study to identify and define candidate data elements [8]. One major advantage of ML techniques is the ability to analyze large datasets for complex relationships among data elements. However, it is essential to validate predictive model accuracy in independent datasets to ensure the generalizability of the model. Even with ML techniques that use rigorous internal cross‐validation methods, predictive ability often degrades or is not generalizable outside of the developing dataset, which is a result of “overfitting” the model. Overfitting, described as occurring when the ML model “is too flexible relative to the data,” is best identified with validation in an independent population. However, some studies or databases available for external validation may not collect the type of data required for accurate clinical prediction. Moreover, there may be differences in patient population characteristics, which sometimes are unmeasurable due to missing data elements [8, 9]. In sum, models for clinical prediction must be validated externally before implementing clinically; for ML techniques, this requires large independent datasets with CDEs and archival data sharing. Pooling data from multiple datasets can also help mitigate the effects of population heterogeneity [9], but, like external validation methods, this requires large datasets with CDEs, which are scarce in maternal health science. Lastly, CDEs could also be used to inform the development of EHRs, with a mind of leveraging EHR‐extracted data for research in the real‐world setting. Implementing a universally accepted set of priority CDEs should be a major advancement in overcoming data‐related barriers to ML‐based disease prediction and prevention. If a universal set of priority CDEs is adopted widely by investigators, we anticipate that data standardization will significantly improve the current suboptimal state of maternal health research, in which collaboration, interpretation, and generalizability are hampered by limitations of data quality and comparability. In turn, improved maternal health science should translate to improved healthcare and health outcomes.

While the development of priority CDEs for maternal health research by Stierman and colleagues is a critical step in harmonizing and sharing clinical research data, realizing meaningful advances in maternal health science and improving maternal health outcomes will require continued effort. Though the need for implementation of priority CDEs is evident, the widespread adoption of these must be approached thoughtfully. Researchers, particularly those with fewer resources, may find adopting these CDEs burdensome, and initial implementation may slow research progress. The planned implementation of these CDEs first at a subset of National Institue of Child Health and Human Development (NICHD)‐funded Implementing a Maternal Health and Pregnancy Outcomes Vision for Everyone (IMPROVE) Maternal Health Research Centers of Excellence will help to refine data element definitions and priorities, assess feasibility, and identify best practices for use [5]; this will ultimately facilitate broader adoption. As the CDEs are more widely implemented, there will undoubtedly be a continued need for refining data element priorities and definitions to ensure that the CDE set remains relevant and useful. Additionally, there is a need to develop priority CDEs for infant health research to complement these maternal health efforts. Finally, as this investigative team correctly asserts, the impact of establishing priority CDEs will only be achieved with earnest efforts in improving data sharing capability and creating common data models.

## CONFLICT OF INTEREST STATEMENT

The authors declare no conflicts of interest.

## FUNDING INFORMATION

The authors received no specific funding for this work.
